# Down-Regulation of MicroRNA-223 Promotes Degranulation via the PI3K/Akt Pathway by Targeting IGF-1R in Mast Cells

**DOI:** 10.1371/journal.pone.0123575

**Published:** 2015-04-13

**Authors:** Quan Wang, De-Yu Zhao, Hong Xu, Hui Zhou, Qian-Yuan Yang, Feng Liu, Guo-Ping Zhou

**Affiliations:** 1 Department of Respiratory Medicine, Nanjing Children’s Hospital Affiliated to Nanjing Medical University, Nanjing, China; 2 Department of Pediatrics, Nanjing Maternal and Child Health Hospital of Nanjing Medical University, Nanjing, China; 3 Department of Pediatrics, the Frist Affiliated Hospital of Nanjing Medical University, Nanjing, China; SAINT LOUIS UNIVERSITY, UNITED STATES

## Abstract

**Background:**

Mast cells play a central role in allergic and inflammatory disorders by inducing degranulation and inflammatory mediator release. Recent reports have shown that miRNAs play an important role in inflammatory response regulation. Therefore, the role of miR-223 in mast cells was investigated.

**Methods:**

The expression of miR-223 was quantified by quantitative real-time polymerase chain reaction (qRT-PCR) in immunoglobulin E (IgE)-mediated mast cells. After successful miR-223 inhibition by transfection, degranulation was detected in IgE-mediated mast cells. The phosphorylation of IκB-α and Akt were examined using western blotting. NF-κB was tested using electrophoretic mobility shift assay. PI3K-inhibitor (LY294002) was used to investigate whether the PI3K/Akt pathway was essential for mast cell activation. The TargetScan database and a luciferase reporter system were used to identify whether insulin-like growth factor 1 receptor (IGF-1R) is a direct target of miR-223.

**Results:**

MiR-223 expression was up-regulated in IgE-mediated mast cells, whereas its down-regulation promoted mast cell degranulation. Levels of IκB-α and Akt phosphorylation as well as NF-κB were increased in miR-223 inhibitor cells. LY294002 could block the PI3K/Akt signaling pathway and rescue the promotion caused by suppressing miR-223 in mast cells. IGF-1R was identified as a direct target of miR-223.

**Conclusions:**

These findings suggest that down-regulation of miR-223 promotes degranulation via the PI3K/Akt pathway by targeting IGF-1R in mast cells.

## Introduction

MicroRNAs (miRNAs) are a class of small non-coding RNAs (approximately 22 nt) that bind to multiple target mRNAs to control gene expression post-transcriptionally by inhibiting translation[1]. These miRNAs are involved in highly regulated processes such as differentiation, proliferation, apoptosis, and metabolic processes[1, 2]. Various studies recently demonstrated that miRNAs also play an important role in regulation of the inflammatory response. For example, MiR-221 regulated the hyperproliferation and interleukin (IL)-6 release of airway smooth muscle cells from patients with severe asthma[3]. Let-7 can reduce IL-13 levels in the lungs and alleviate both airway hyper-responsiveness and airway inflammation in a murine model of asthma[4]. Among the known miRNAs, miRNA-223 has gained more attention in inflammation. Studies found that miR-223 overexpression inhibited IL-1β production from the inflammasome[5]. MiR-223 was critical for the control of tuberculosis and potentially other chronic inflammatory diseases by regulating leukocyte chemotaxis via chemoattractants[6]. Moreover, miR-223 can suppress the proinflammatory activation of macrophages[7]. While the inflammation of miRNA-223 in various cells and diseases is well established by now, very little is known about the role of miRNA-223 in mast cells.

Mast cells play a crucial role in the initiation of the inflammatory reactions that are associated with allergic disorders, such as asthma, atopic dermatitis, and rheumatic synovitis[8, 9]. Mast cells express high-affinity Fc epsilon RI (FcεRI), which binds IgE to induce mast cell activation[10]. Aggregation of FcεRI by polyvalent antigen leads mast cells to degranulation and the secretion of histamine, cytokines, and other chemical mediators. Downstream signaling is largely dependent on the different isoforms of the phosphoinositide-3-kinase (PI3K) family members[11]. However, the signaling pathways of degranulation are complicated and not fully understood.

In the present study, miR-223 expression was up-regulated in IgE-mediated mast cells. The effect of miR-223 on IgE-mediated degranulation and the potential mechanism were investigated. Finally, our findings suggest that down-regulation of miR-223 promotes degranulation via the PI3K/Akt pathway by targeting insulin-like growth factor 1 receptor (IGF-1R) in mast cells.

## Materials and Methods

### Cell culture

The mast cell line RBL-2H3 was obtained from the Cell Resources Center of Shanghai Institutes for Biological Sciences, Shanghai, China. The cells were maintained in Eagle’s minimum essential medium containing 10% fetal bovine serum (Gibco BRL, Grand Island, NY, USA) in a humidified atmosphere of 5% CO_2_ at 37°C.

### Quantitative real-time polymerase chain reaction (qRT-PCR) for miRNA-223 in IgE-mediated mast cells

After 24 h of seeding in 6-well tissue culture plates, RBL-2H3 cells were sensitized with 250 ng/mL anti-2,4-dinitrophenyl (DNP) IgE (Sigma-Aldrich) overnight. The cells were then washed twice in Tyrode’s buffer (135 mM NaCl, 5 mM KCl, 1.8 mM CaCl_2_, 1.0 mM MgCl_2_, 5.6 mM glucose, 20 mM HEPES, and 1 mg/mL bovine serum albumin at pH 7.4) and triggered with or without 100 ng/mL DNP–human serum albumin (HSA) (Sigma-Aldrich) for 4 h. After stimulation, total RNA was purified from cells using TRIzol Reagent (Invitrogen). To analyze miRNA-223 expression, qRT-PCR was performed using an miRNA reverse transcription kit and TaqMan miRNA assays from Applied Biosystems according to the manufacturer’s instructions.

### Transfection of miR-223 inhibitor

RBL-2H3 cells were transfected using Lipofectamine 2000 (Invitrogen) the day after cell seeding in accordance with the manufacturer’s instructions. The miR-223 inhibitor (Invitrogen) and its control (Invitrogen) were used at a final concentration of 100 nM. At 24 h post-transfection, follow-up experiments were performed.

### Measurement of degranulation

After 24 h of transfection, the cells were sensitized with 250 ng/mL anti-DNP IgE overnight and washed twice in Tyrode’s buffer before being stimulated with 100 ng/mL DNP-HSA for 1 h. Degranulation reactions were stopped by placing the cells on ice for 10 min. To determine the amount of β-hexosaminidase released by the cells, 50 μL of the supernatants and 50 μL of 1 mM 4-nitrophenyl N-acetyl-β-D-glucosaminide in 50 mM citric acid (pH 4.4) was mixed in separate 96-well plates and incubated at 37°C for 1 h. The reactions were terminated by the addition of 200 mL of carbonate buffer (100 mM Na_2_CO_3_ and 100 mM NaHCO_3_). Cell pellets were lysed in Tyrode’s buffer with 1% Triton X-100 and incubated in citrate buffer to measure total β-hexosaminidase content. Absorbance was read at 405 nm using an enzyme-linked immunosorbent assay plate reader and release of β-hexosaminidase was expressed as fraction of the total enzyme found in unstimulated cells.

### Western blotting

After 24 h of transfection, the cells were sensitized as described above and stimulated for 10 min. Whole-cell lysates were separated on 10% sodium dodecyl sulfate–polyacrylamide gel electrophoresis and transferred to a nitrocellulose membrane. The membranes were blocked with 5% non-fat dry milk in Tris-buffered saline and incubated with anti-phospho-AKT, anti-AKT, anti-phospho-IκB-α, anti-IκB-α (Cell Signaling Technology Inc., Danvers, MA, USA), or anti-IGF-1R (Santa Cruz, Dallas, Texas, USA) antibodies overnight. After washing, the membranes were incubated with a horseradish peroxidase–conjugated secondary antibody and then developed using the enhanced chemiluminescence detection system. Densitometric values of the phosphoprotein bands were normalized against the corresponding total protein.

### Electrophoretic mobility shift assay (EMSA)

The cells were transfected and sensitized as described above and then stimulated for 10 min. The EMSA kit was procured from Pierce (Rockford, IL, USA), and 5'-biotin–labeled NF-κB oligo 5′-AGTTGAGGGGACTTTCCCAGGC-3′ was purchased from Sigma-Aldrich. The biotin end-labeled probe was incubated with nuclear extracts (10 μg) and then electrophoresed on a 6% polyacrylamide gel. Following the electrophoretic transfer of the bound complexes to a nylon membrane, the transferred DNA was cross-linked to the membrane. Biotin-labeled DNA was detected through chemiluminescence using ChemiDoc XRS+ System with Image Lab Software.

### Luciferase reporter assay

Mast cells were seeded in 24-well tissue culture plates the day before transfection. The cells were then co-transfected with IGF-1R-3′UTR vector and miR-223 mimics (Invitrogen) or miR-223 inhibitor. After 24 h of transfection, the lysates were harvested and the luciferase activities were measured using the Dual Luciferase Reporter Assay kit (Promega, Madison, WI, USA).

### Statistical analysis

All data are presented as mean ± SD. Statistical significance of the data was determined by Student’s *t* test utilizing the SPSS 20.0 version. Differences between groups were considered statistically significant at *p* values < 0.05.

## Results

### MiR-223 is up-regulated in IgE-mediated mast cells

**Fig 1:**
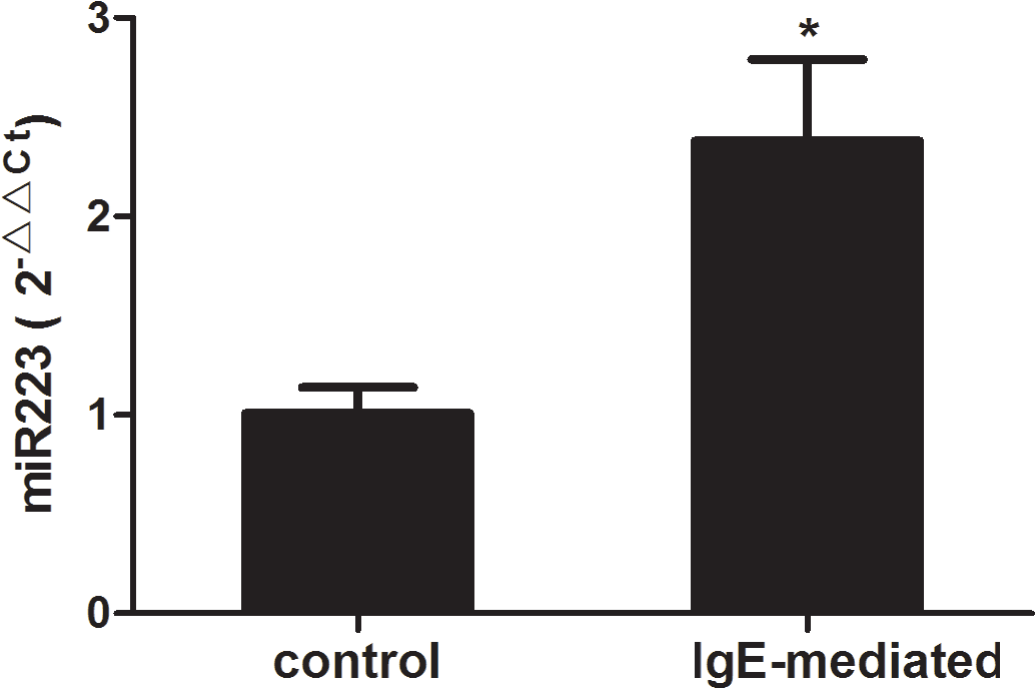
MiRNA-223 is up-regulated in immunoglobulin E (IgE)-mediated mast cells. RBL-2H3 cells were sensitized with anti-2,4-dinitrophenyl (DNP) IgE and then triggered with (IgE-mediated group) or without (control group) DNP–human serum albumin for 4 h. The expression of miR-223 were tested by qRT-PCR using the 2^-ΔΔCt^ method. Data are represented as the mean ± SD for three experiments (n = 4). *P < 0.01 compared to the control group.

To investigate the role of miR-223 in mast cells, miR-223 expression in the cells was first evaluated. RBL-2H3 cells were sensitized with anti-DNP IgE and then triggered with (IgE-mediated group) or without (control group) DNP-HSA for 4 h. MiR-223 expression was tested by qRT-PCR in the two groups. Compared to the control group, IgE-mediated mast cells displayed significantly higher miR-223 expression (Fig 1).

### Down-regulation of miR-223 promotes degranulation

**Fig 2:**
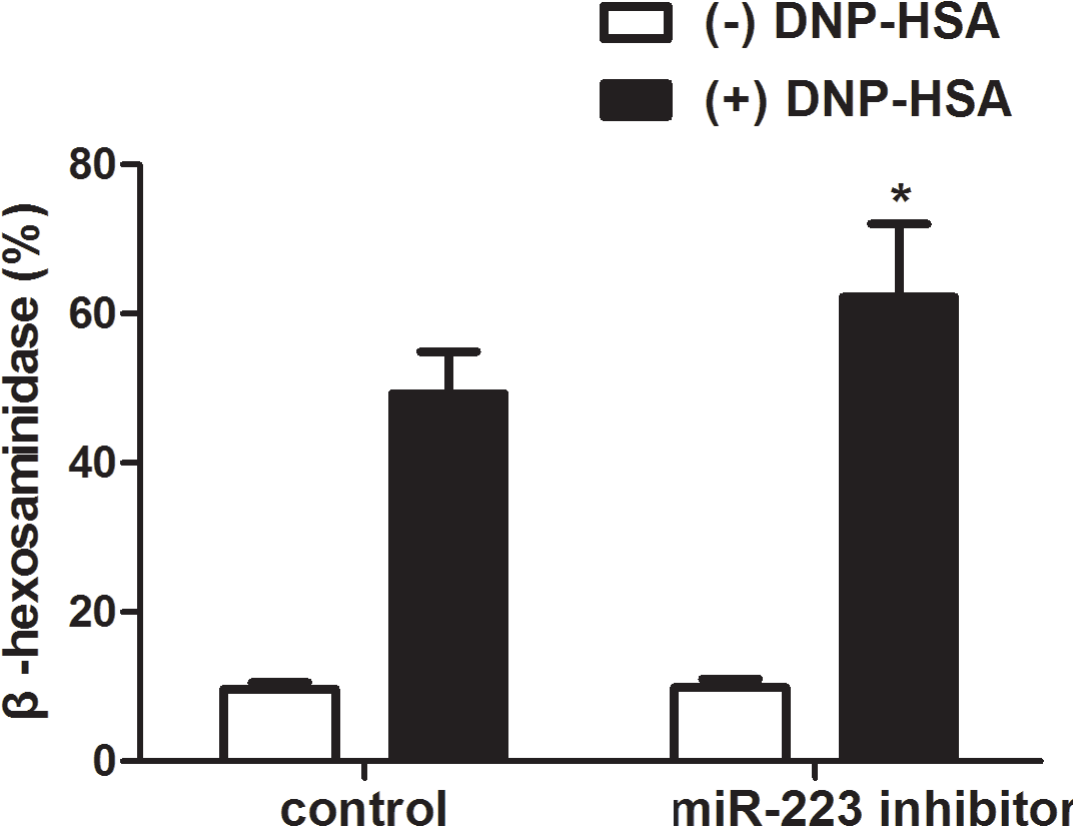
Down-regulation of miR-223 promotes degranulation. Mast cells were transfected with miR-223 inhibitor (miR-223 inhibitor group) or its control (control group). The cells were then sensitized with anti-2,4-dinitrophenyl (DNP) immunoglobulin E and triggered with (black column) or without (white column) DNP-HSA. After triggering for 1 h, release of β-hexosaminidase were detected in the two groups. Data are represented as the mean ± SD for three experiments (n = 6). *P < 0.05 compared to the control group.

Since the expression of miR-223 was higher in IgE-mediated mast cells, the function of miR-223 was inhibited using a miR-223–specific inhibitor. The cells were transfected with the miR-223 inhibitor (miR-223 inhibitor group) or its control (control group). The cells were then sensitized with anti-DNP IgE and triggered with or without DNP-HSA for 1 h. The degranulation (release of β-hexosaminidase) were next detected in the two groups. Compared to the control group, the miR-223 inhibitor group showed significantly higher β-hexosaminidase release (Fig 2).

### Effect of miR-223 on the PI3K/Akt pathway

**Fig 3:**
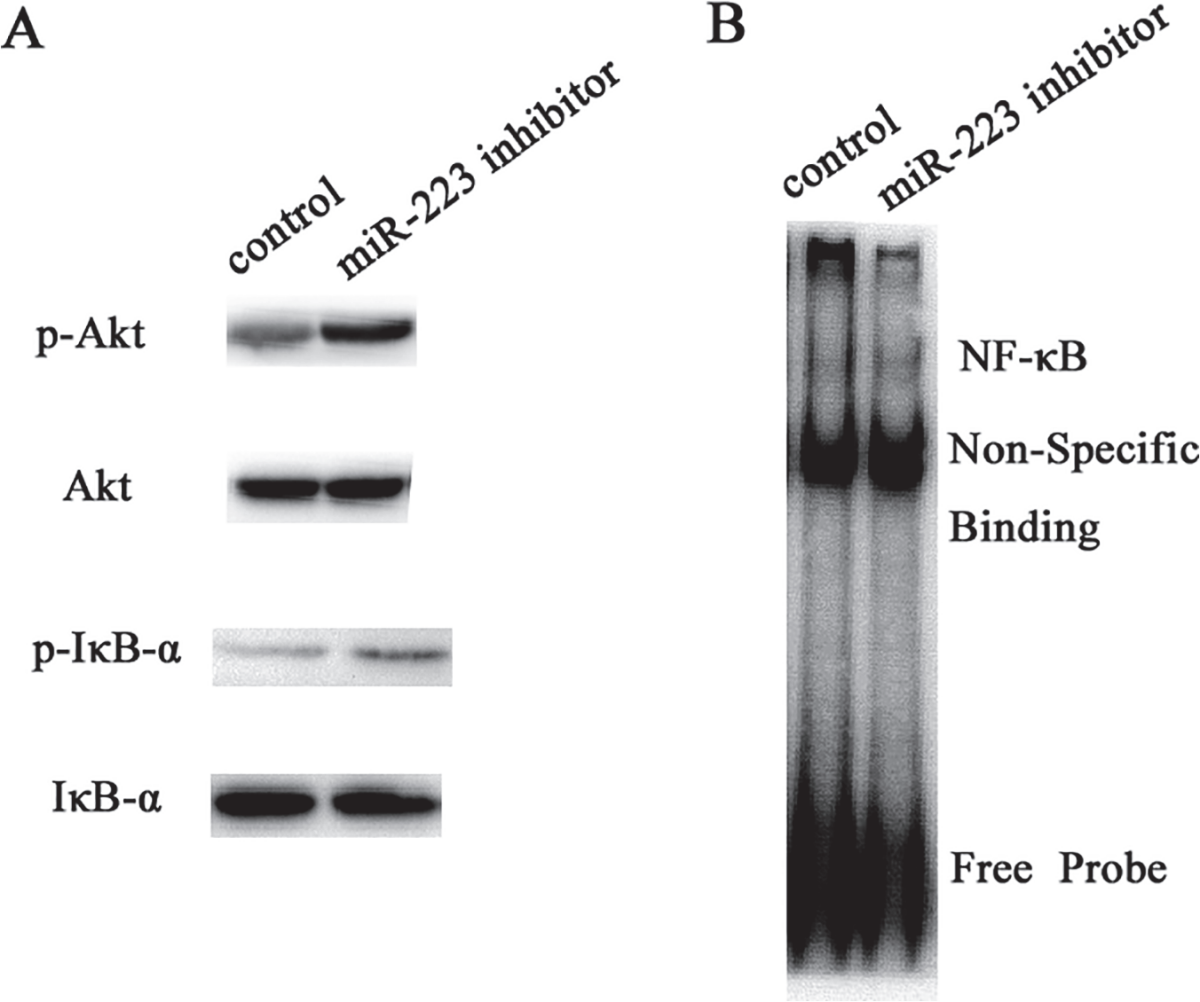
Effect of miR-223 on PI3K/Akt pathway. (A) The cells were transfected with miR-223 inhibitor (miR-223 inhibitor group) or its control (control group). The cells were then sensitized with anti-2,4-dinotrophenyl (DNP) immunoglobulin E (IgE) and were triggered with DNP–human serum albumin for 10 min. Whole-cell lysates were used to examine the phosphorylation of IκB-α and Akt and their total protein levels by western blotting. (B) Nuclear extracts were used to test NF-κB by EMSA.

Mast cell activation is controlled by the PI3K/Akt pathway and the downstream signaling processes. To determine the mechanism by which miR-223 affects mast cell degranulation, the cells were transfected with miR-223 inhibitor (miR-223 inhibtor group) or its control (control group). The cells were then sensitized with anti-DNP IgE and triggered with DNP-HSA for 10 min. Whole-cell lysates were used to examine the phosphorylation of IκB-α and Akt and their total protein levels by western blotting. Nuclear extracts were used to test NF-κB by EMSA. However, the levels of IκB-α and Akt phosphorylation and NF-κB were increased in the miR-223 inhibitor group compared to the control group (Fig 3A and 3B). The enhanced non-site-specific Akt phosphorylation, which is considered a surrogate marker for PI3K activity, strongly suggests that the PI3K pathway is negatively regulated by miR-223.

### PI3K-inhibitor (LY294002) decreases degranulation

**Fig 4:**
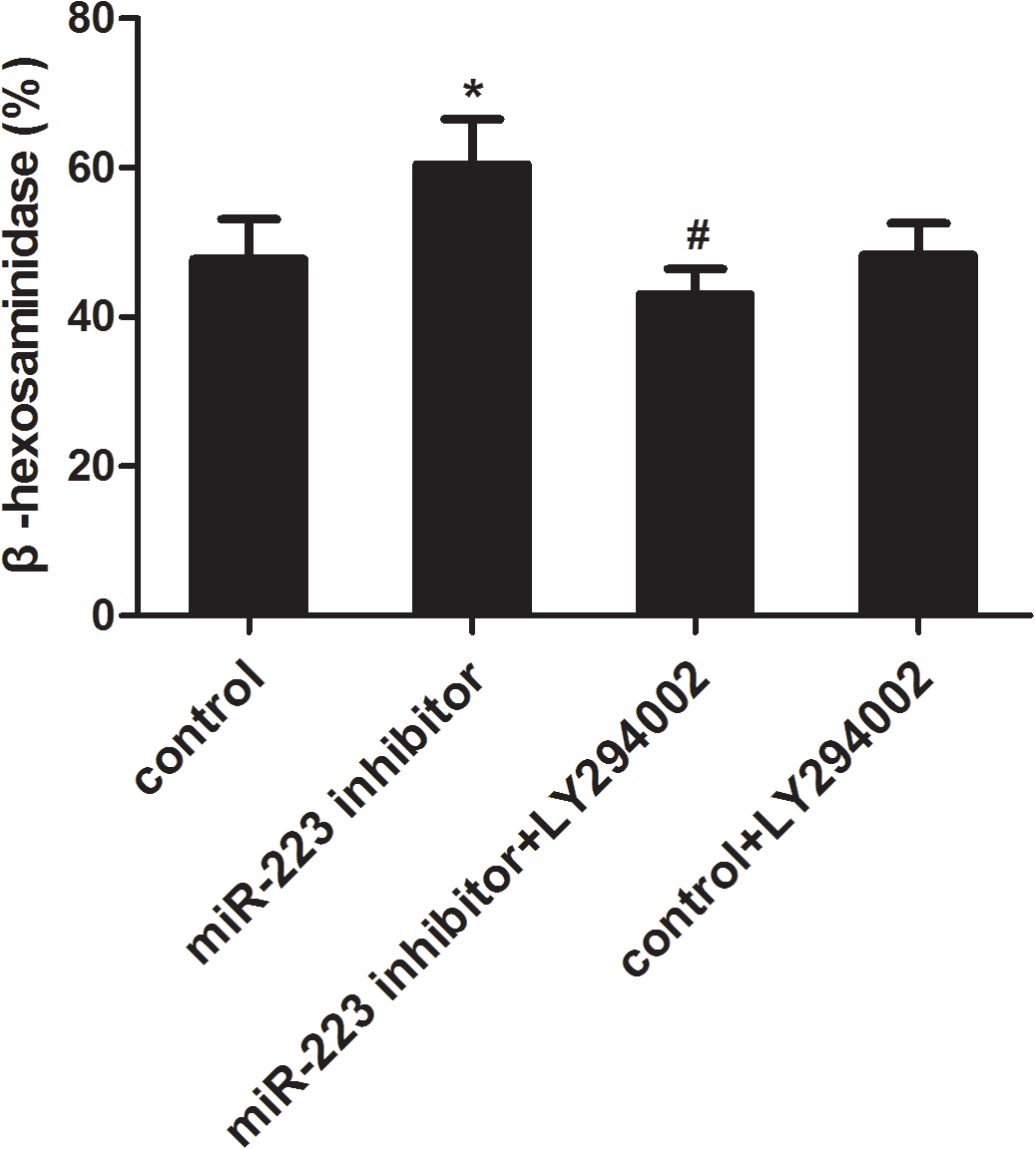
PI3K-inhibitor (LY294002) decreases degranulation. The cells were transfected with miR-223 inhibitor or its control and were sensitized with anti-DNP IgE overnight. The cells were then triggered with DNP-HSA with or without LY294002. After triggering for 1 h, release of β-hexosaminidase were detected in four groups. Data are represented as the mean ± SD for three experiments (n = 6). *P < 0.01 compared to the control group; ^#^ *P* < 0.01 compared to the miR-223 inhibitor group.

To investigate whether the PI3K/Akt signaling pathway is essential for mast cell degranulation, the specific PI3K inhibitor (LY294002) was used to intervene the cells. The cells were transfected with miR-223 inhibitor or its control and sensitized with anti-DNP IgE overnight. Then the cells were triggered with DNP-HSA with or without LY294002. After triggering for 1 h, β-hexosaminidase release was detected in the four groups. Compared to the miR-223 inhibitor group, β-hexosaminidase release significantly reduced in the miR-223 inhibitor + LY294002 group. Consistent with the previous result, the miR-223 inhibitor group showed significantly higher β-hexosaminidase release compared to the control group. However, β-hexosaminidase release in the control group and the control + LY group showed no statistically significant difference (Fig 4). In summary, LY294002 could block the PI3K/Akt signaling pathway and rescue the promotion caused by suppressing miR-223 on mast cells.

### IGF-1R is targeted by miR-223

**Fig 5:**
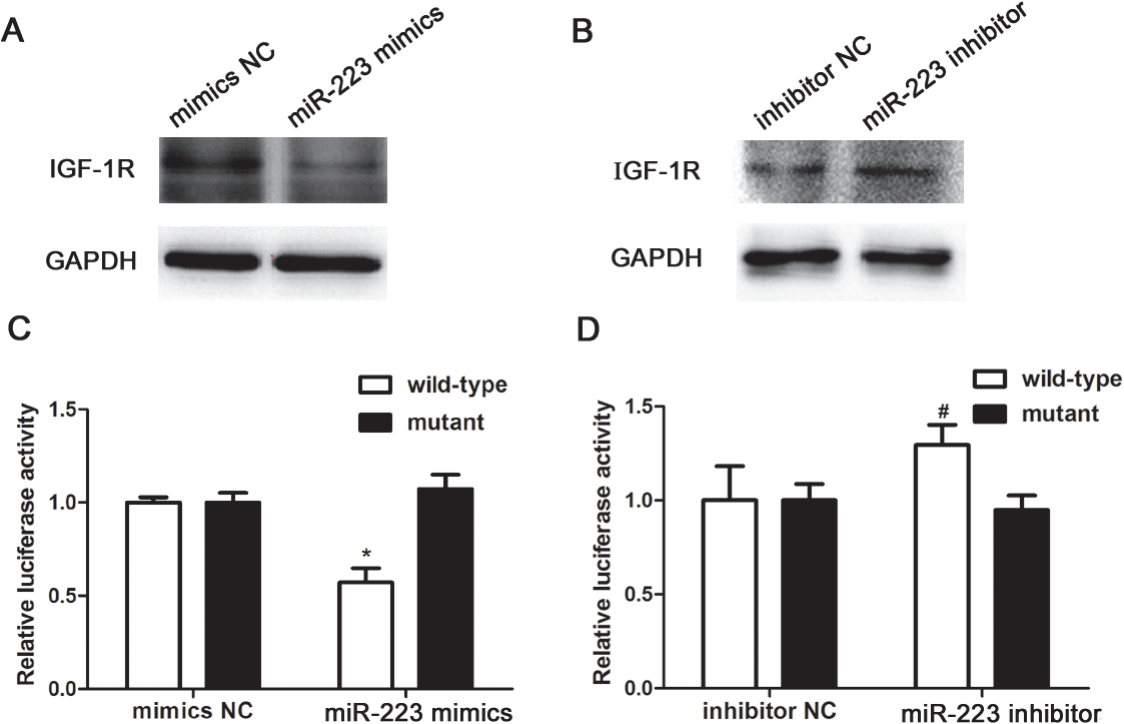
Insulin-like growth factor 1 receptor (IGF-1R) is targeted by miR-223. (A and B) The cells were transfected with miR-223 mimics (mimics NC as control) or miR-223 inhibitor (inhibitor NC as control). At 24 h post-transfection, the expression of IGF-1R were determined by western blotting. (C and D) To assess whether IGF-1R is a direct target of miR-223, the cells were co-transfected with IGF-1R-3′UTR vector and miR-223 mimics (mimics NC as control) or miR-223 inhibitor (inhibitor NC as control). After 24 h of transfection, the lysates were harvested and luciferase activities were measured. Data are represented as the mean ± SD for three experiments (n = 4). *P < 0.01 compared to the mimics NC group; ^#^ *P* < 0.05 compared to the inhibitor NC group.

The function of miRNA primarily relies on its target genes. To determine the further mechanism of miR-223 in mast cells, miR-223’s target gene was searched using the public database TargetScan (http://www.targetscan.org), and IGF-1R that possessed a critically conserved binding site for miR-223 was selected for further molecular and functional confirmation. To investigate whether miR-223 expression was correlated with IGF-1R protein expression, the cells were transfected with miR-223 mimics (mimics NC as control) or miR-223 inhibitor (inhibitor NC as control). At 24 h post-transfection, the expression of IGF-1R was determined by western blotting. The results showed that IGF-1R significantly decreased in the miR-223 mimics group while it increased in the miR-223 inhibitor group (Fig 5A and 5B). To assess whether IGF-1R was a direct target of miR-223, the cells were co-transfected with IGF-1R-3′UTR vector and miR-223 mimics (mimics NC as control) or miR-223 inhibitor (inhibitor NC as control). After 24 h of transfection, the lysates were harvested and the luciferase activities were measured. Cells transfected with miR-223 mimics decreased luciferase activity, but when IGF-1R-3′UTR was mutated, there was no decrease in luciferase activity. Although reduction of miR-223 expression by transfecting miR-223 inhibitor into mast cells resulted in an induction of luciferase activity, IGF-1R-3′UTR mutation had no effect on luciferase activity (Fig 5C and 5D).

## Discussion

MiRNAs are becoming hotspots for their multiple biological functions. Among them, miRNA-223 can regulate cell differentiation, proliferation, and apoptosis[12–14]. Furthermore, recent compelling studies suggested a significant role of miR-223 in inflammation. Evidence showed that miR-223 overexpression inhibited IL-1β production from the inflammasome[5]. MiR-223 was critical for the control of tuberculosis and potentially other chronic inflammatory diseases by regulating leukocyte chemotaxis via chemoattractants[6]. Moreover, miR-223 can suppress the proinflammatory activation of macrophages[7]. In our experiments, miR-223 expression was higher in IgE-mediated mast cells. Down-regulation of miR-223 promoted degranulation.

Mast cells are the most important effector cells in IgE-mediated allergic immune responses[15]. The mediator secreted from mast cells is controlled by FcεRI-dependent signaling pathways. Induction of second messengers and PKC activation by proximal FcεRI signaling events have been intensely studied, whereas coupling of these signals to exocytosis of mast cell granules is incompletely understood. Research has shown that IκB kinase2 was a central player in mast cell degranulation by phosphorylating the SNARE protein SNAP23[16]. However, Katrin et al. reported that IκB kinase 2 was dispensable for mast cell degranulation[17]. In this study, miR-223 promoted degranulation by regulating the PI3K/Akt pathway and downstream NF-κB and IκB-α signaling was determined. Importantly, LY294002 could rescue the promotion caused by suppressing miR-223 in mast cells, suggesting PI3K/Akt signaling pathway was essential for mast cell degranulation. Coincidentally, miRNA-155 also controlled mast cell degranulation by regulating the PI3K/Akt pathway[18].

The IGF-1R signaling pathway is involved in normal cell growth and development. Much evidence has shown that miR-223 regulates cell differentiation, proliferation, and apoptosis by targeting IGF-1R[12–14]. IGF-1R can also activate many downstream kinases, including Akt, Erk, and Ampk[19]. Furthermore, Akt plays an important role in inflammatory reactions. IGF-1R was targeted by miR-223 in this study.

Based on the results of the present study, it appears that down-regulation of miR-223 promotes degranulation via the PI3K/Akt pathway by targeting IGF-1R in mast cells.

## Supporting Information

S1 File The data of Fig 1. (XLS)

S2 File The data of Fig 2. (XLS)

S3 File The data of Fig 4. (XLS)

S4 File The data of Fig 5C and 5D. (XLS)
